# Textured Lead-Free Piezoelectric Ceramics: A Review of Template Effects

**DOI:** 10.3390/ma18030477

**Published:** 2025-01-21

**Authors:** Temesgen Tadeyos Zate, Cenk Abdurrahmanoglu, Vincenzo Esposito, Astri Bjørnetun Haugen

**Affiliations:** Department of Energy Conversion and Storage, Technical University of Denmark, Anker Engelunds vej, Building 301, 2800 Kgs Lyngby, Denmark

**Keywords:** piezoceramics, crystallographic texture engineering, templated grain growth, template morphology, template composition, template crystallographic orientation

## Abstract

Crystallographic texture engineering through templated grain growth (TGG) has gained prominence as a highly effective strategy for optimizing the electromechanical performance of lead-free piezoelectric ceramics, offering a pathway toward sustainable alternatives to lead-based systems like lead zirconate titanate (PZT). By achieving high degrees of texture, with Lotgering factors (LFs) often exceeding 90%, these systems have demonstrated piezoelectric properties that rival or even surpass their lead-based counterparts. Despite these advancements, the field lacks a comprehensive understanding of how specific template parameters influence the texture quality and functional properties across different material systems. This review provides an in-depth analysis of the influence of the template morphology, composition, and crystallographic orientation on the texturing of key lead-free systems, including BaTiO_3_ (BT), (K_0.5_Na_0.5_)NbO_3_ (KNN), and Bi_0.5_Na_0.5_TiO_3_ (BNT). Furthermore, it explores how the template selection affects the induced crystallographic direction, and how this impacts the material’s phase structure and domain configurations, ultimately influencing the piezoelectric and dielectric properties. By consolidating the existing knowledge and identifying current challenges, this work highlights key strategies for optimizing the texture and electromechanical performance in lead-free ceramics, providing essential insights for future research aimed at advancing high-performance, environmentally friendly piezoelectric materials for applications such as sensors, actuators, and energy-harvesting devices.

## 1. Introduction

Piezoelectric ceramics are integral to a wide range of applications, including actuators, sensors, and energy harvesters, due to their ability to interconvert mechanical and electrical energy [1]. For decades, lead-based materials like lead zirconate titanate (PZT) have been the benchmark for high-performance piezoelectric devices, attributed to their superior piezoelectric characteristics. The commonly used PZT compositions are based on PbZr_0.5_Ti_0.5_O_3_ (with small amounts of proprietary and application-specific dopants such as Fe, La, or Nb), resulting in a lead oxide content of up to 70 wt% [2]. Increasing concerns over environmental and health hazards posed by lead pollution have therefore driven significant research into lead-free alternatives, reflecting the urgent need for more sustainable materials in the field [3].

Among the most promising candidates for lead-free piezoelectric ceramics, potassium sodium niobate (KNN)-, bismuth sodium titanate (BNT)-, and barium titanate (BT)-based systems have attracted considerable attention [4]. However, despite their potential, these materials often fail to achieve the performance levels of their lead-based counterparts [5]. Several strategies have been explored to bridge the performance gap, including chemical substitution, doping, microstructural control, domain engineering, phase boundary engineering, and crystallographic texturing [4,5,6]. Among these approaches, crystallographic texturing via templated grain growth (TGG) has emerged as a particularly effective method for enhancing the piezoelectric properties in both lead-based and lead-free systems [6,7,8,9,10,11]. TGG facilitates grain alignment along favorable crystallographic orientations, optimizing the intrinsic crystallographic properties and extrinsic structural features. By achieving a high degree of texture, ceramics produced via TGG exhibit properties that not only approach but, in some cases, surpass those of the traditional nontextured lead-based ceramics [12,13,14]. This advancement presents a promising pathway for developing high-performance, lead-free materials. While the piezoelectric performance of lead-free textured ceramics generally remains below that of their lead-based textured counterparts, they still have a sufficient performance to replace the traditional nontextured, lead-based piezoelectrics in many applications. Compared to single crystals, textured ceramics are a more cost-efficient approach with less material chemistry limitations, and piezoelectric responses above 70% of the single-crystal values are commonly obtained [6].

The TGG process relies on templates. These are large anisometric particles, usually between 5 and 15 µm, typically exhibiting plate-like or needle-like morphologies [7,8,15,16]. A unique crystallographic direction, generally (001) parallel to the needle length or [001] plate normal, is ensured by the templates being either single crystals or aggregates of aligned polycrystals. These templates act as nucleation sites that direct the orientation of ceramic grains during subsequent growth. The careful selection and preparation of these templates are critical, as their morphology and crystallographic orientation directly influence the final texture of the ceramic and its functional properties. Ideal templates should be highly anisometric, have a crystal structure compatible with the matrix material (the lattice mismatch should be below 15%), and be chemically stable during the sintering process [6,7,10,16,17]. Templates are typically synthesized by molten salt or hydrothermal processes. Here, their anisometric shapes can freely develop due to the different directional growth rates inherent to their crystal structures, or they can be a product of a topochemical conversion from another anisometric particle [6]. Both 1D (rod) and 2D (plate) templates can be made with these techniques.

**Figure 1:**
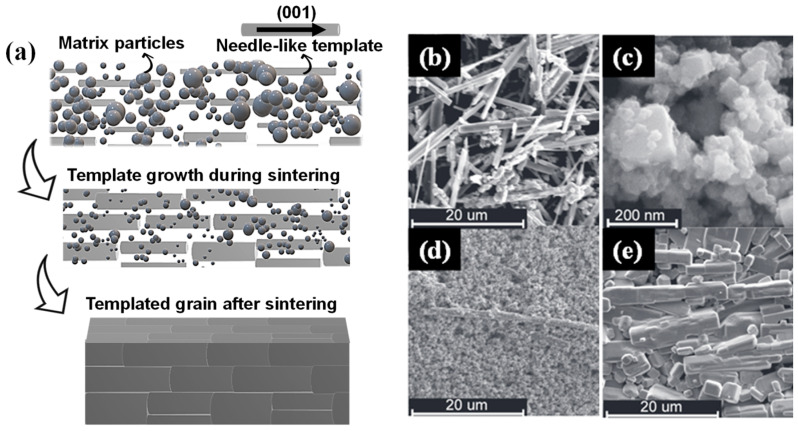
(**a**) Schematic illustration of the standard TGG method. (**b**) SEM image of needle-like KNN templates prepared for tape casting. (**c**) Submicron KNN matrix powder used in tape casting. (**d**) Green tape with aligned KNN templates in the KNN matrix after tape casting. (**e**) Final sintered compact showing textured KNN ceramics with highly oriented grains. Figures (**b**–**d**) are reprinted with permission from [18].

To address the diverse material and process requirements in developing textured lead-free piezoelectric ceramics, the TGG method has evolved into several variants [8,15]. The standard TGG approach employs homoepitaxial templates, which share the same chemical composition as that of the matrix, ensuring compatibility and minimal lattice mismatch. As illustrated in Figure 1a–e, this process begins with the alignment of needle-like or plate-like templates (Figure 1b) within a submicron powder matrix (Figure 1c), forming green tapes with well-oriented templates post-tape-casting (Figure 1d). During sintering, the templates guide the grain growth, resulting in a highly oriented microstructure, as observed in the sintered compacts of textured KNN ceramics using KNN needle-like templates [18] (Figure 1e).

**Figure 2:**
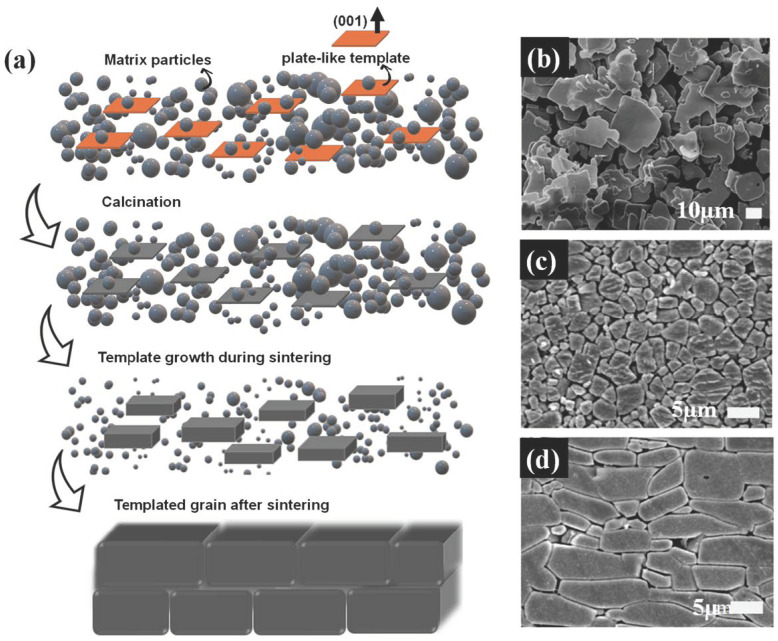
(**a**) Schematic representation of the RTGG process, highlighting the chemical reaction during calcination that results in a stoichiometric composition between the template and matrix. (**b**) SEM image of BNT15 templates with a plate-like morphology. (**c**) Microstructure of nontextured BNKT ceramics for comparison. (**d**) Textured BNKT ceramics obtained by RTGG using 3 mol% BNT15 templates. Figures (**b**–**d**) are reprinted with permission from [19].

In contrast, reactive TGG (RTGG) incorporates reactive templates with non-stoichiometric matrix compositions. During calcination, these templates undergo chemical reactions with the matrix, as depicted in Figure 2a [15]. These reactions result in a stoichiometric and chemically identical composition between the template and matrix. For example, BNT15 templates (Figure 2b) react with a BNKT matrix to form the same composition [19]. The resulting textured brick-like microstructure is shown in Figure 2d compared to nontextured BNKT ceramics (Figure 2c).

**Figure 3:**
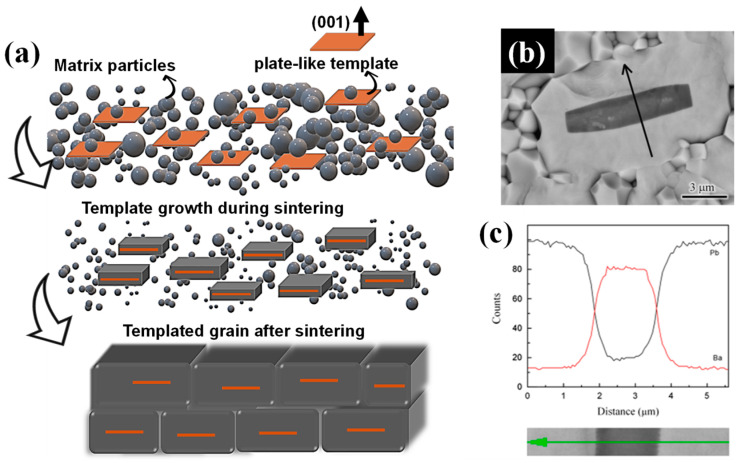
(**a**) Schematic of the HTGG process with chemically distinct BT templates embedded in a PMN-PT matrix. (**b**) SEM image showing BT templates within the PMN-PT matrix after sintering. (**c**) EDS line scan across a BT template, illustrating the elemental distribution and confirming the interaction between the BT template and the PMN-PT matrix without compromising the core structure. Figures (**b**,**c**) are reprinted with permission from [20]. The arrow in (**b**,**c**) indicates the EDS scan direction.

Heteroepitaxial TGG (HTGG), in contrast, employs chemically distinct templates when homoepitaxial options are unavailable. These hetero-templates grow by consuming finer matrix particles during sintering, promoting grain orientation while retaining their core structure. An example is the use of BT templates in a PMN-PT matrix [20], as illustrated in Figure 3a–c. Here, the BT template aligns within the PMN-PT matrix, and the elemental distribution across the template, captured via EDS line scanning (Figure 3c), confirms that the grain growth occurs without a detectable chemical reaction between the template and matrix, preserving the core structure of the template.

Template alignment within the ceramic matrix is achieved through various techniques, including tape casting, screen printing, and more recently, vat photopolymerization (VPP) and electrophoretic deposition (EPD) [6,10,11,16,21,22,23]. Among these, tape casting remains the most established and widely used method [10]. Templates of any crystallographic orientation can be used; however, most studies use (001)-oriented platelets to generate ceramics with a [001] texture perpendicular to the tape-cast plane, usually without justifying this choice of crystallographic direction.

Several techniques can be used to characterize the degree of texture, for example, X-ray diffraction and electron backscatter diffraction [18]. By far, the most commonly used quantification is the Lotgering factor (LF) [6]. Here, the ratio of the intensity of the diffraction peaks in the textured direction relative to all the other crystallographic directions in a textured sample are compared to a nontextured reference sample of the same composition:

$$LF=\left(P_{\left(00l\right)}-P_{o}\right)/\left(1-P_{o}\right)$$

$$P_{\left(00l\right)}=\sum I_{\left(00l\right)}/\sum I_{\left(hkl\right)}$$

$$P_{o}=\sum I_{o\left(00l\right)}/\sum I_{o\left(hkl\right)}$$

where *P*_(00*l*)_ and *P*_0_ are the sums of the XRD peak intensities of all (00*l*) peaks divided by all (hkl) (*hkl*) peaks of textured ceramics and nontextured ceramics, respectively. This gives a fraction (or percentage) of the alignment, which is easily obtained and comparable between studies, but it only accounts for grains perfectly aligned in the texture direction [6].

**Figure 4:**
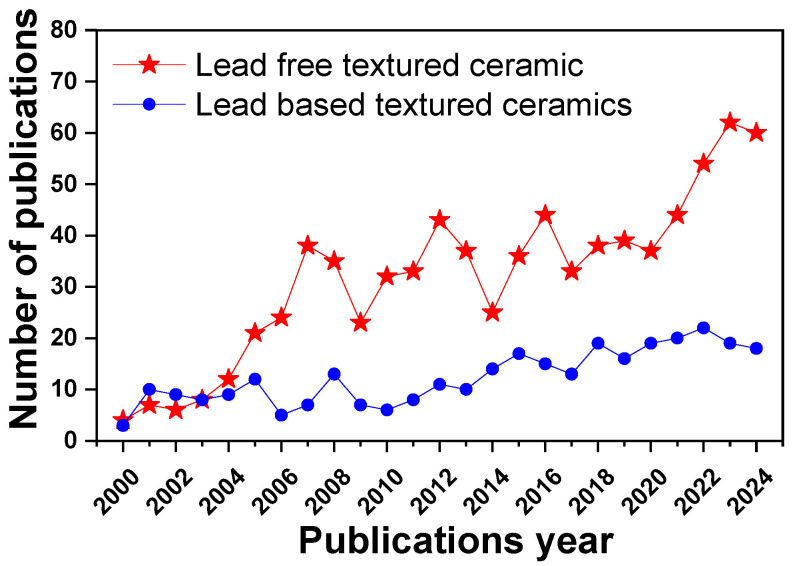
Comparison of the number of publications on lead-free and lead-based textured ceramics from 2000 to 2024 (Web of Science accessed on 15 December 2024, https://www.webofscience.com).

Previous reviews have significantly contributed to advancing this field [6,7,8,9,10,11,15,24,25,26]. Messing et al. [6] provide instrumental principles in elucidating texture engineering and its historical context, while Zhang et al. [7] provide a comprehensive overview of textured ceramics. Chang et al. [8] briefly address the importance of template selection in piezoceramics, and Moriana et al. [10] address lead-free textured ceramics using the tape-casting method. Additionally, Walton et al. [11] explored the potential of the additive manufacturing of textured ceramics. While these studies have laid a strong foundation, a detailed investigation into template selection strategies to achieve the optimal performance in texturing lead-free systems is still lacking. Given the rapid growth of the research and development of lead-free textured ceramics compared to lead-based systems (Figure 4), it is essential to comprehensively understand how templates influence the texture and overall material properties. This is because the choice of the template directly governs the crystallographic texture, significantly impacting textured ceramics’ performance. Furthermore, when comparing textured ceramics with conventional polycrystalline ceramics, the templates constitute a new material that needs to be acquired, either through in-house synthesis or from one of the few commercially available sources.

**Figure 5:**
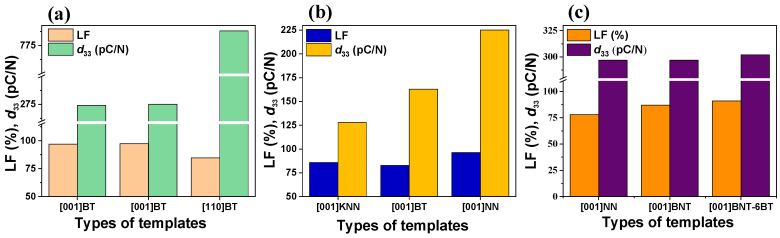
Lotgering factor (LF) and piezoelectric charge coefficient (*d*_33_) of (**a**) textured BaTiO_3_ (BT) ceramics with (001) and (110) BT templates, (**b**) textured (K,Na)NbO_3_ (KNN) ceramics with (001) KNN, (001) BT, and (001) NN templates, and (**c**) textured BNT-6BT ceramics with (001) NN, (001) BNT, and (001) BNT-6BT templates.

For instance, as shown in Figure 5a, BaTiO_3_ (BT) ceramics textured with a (001)-oriented template achieved piezoelectric charge coefficient (*d*_33_) values of 275 pC/N and 274 pC/N, twice as much as the nontextured BT, as reported by Du et al. [27] and Vriami et al. [21], respectively. Both studies also reported high degrees of texture, with Lotgering factors (LFs) exceeding 95%. However, Wada et al. [12] demonstrated that BT ceramics textured with a (110)-oriented template achieved a much higher *d*_33_ of 788 pC/N, despite a slightly lower LF of 84.5%. These results highlight that while a high degree of texture is beneficial, the specific crystallographic orientation of the template plays a critical role in determining the material’s performance. This effect further corroborates the importance of carefully selecting the template, as its orientation directly and profoundly influences the piezoelectric and overall functional properties of the textured ceramics.

Similarly, in Figure 5b, textured K_0.5_Na_0.5_NbO_3_ (KNN) ceramics show varying *d*_33_ values when templates of different compositions were used: 128 pC/N using (001) KNN templates with an LF of 86% [18], 163 pC/N using a (001) BT template with an LF of 80% [28], and 225 pC/N using a (001) NaNbO_3_ (NN) template with an LF of 96.2% [29]. This variation highlights the significant impact of the template chemistry on the degree of texture and piezoelectric performance of textured ceramics. Conversely, as seen in Figure 5c, textured Bi_0.5_Na_0.5_TiO_3_-6%BT (BNT-6BT) ceramics exhibit relatively consistent *d*_33_ values around 300 pC/N, irrespective of whether (001) NN [30], (001) BNT [31], or (001) BNT-6BT [32] templates were used. This result suggests that for BNT-6BT ceramics, the template chemistry is less influential in determining the *d*_33_ values; rather, the impact is seen in the dielectric properties, which will be discussed later in Section 4.1.5. In addition to the template orientation and chemistry, the shape and size of the templates play a crucial role in shaping the final microstructure, which subsequently influences the performance of the textured ceramics [10,33,34]. Therefore, this review aims to consolidate the existing knowledge on the role of templates in the development of textured lead-free ceramics, focusing on how the template composition, morphology, and orientation influence the crystallographic control and piezoelectric performance in KNN-, BNT-BT-, and BT-based materials, ultimately guiding future research in this promising field.

## 2. Textured BaTiO_3_-Based Ceramics

**Figure 6:**
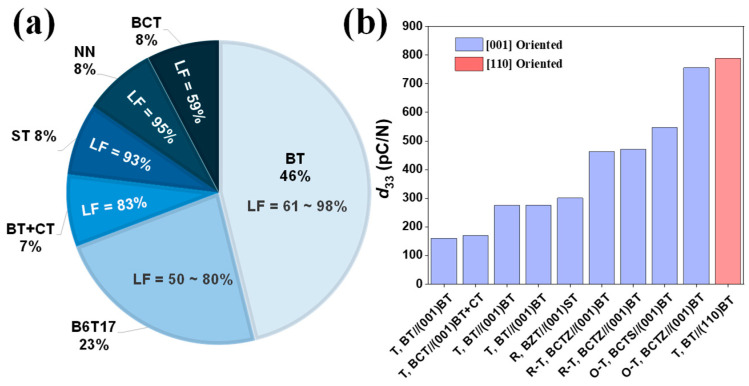
(**a**) Template distribution in textured BT-based ceramics. Lotgering factor (LF) represents the degree of texture in percent. (**b**) Piezoelectric charge coefficient (*d*_33_) values of various textured BT-based ceramics. T, R, and O represent tetragonal, rhombohedral, and orthorhombic phases. Expressions like “T, BT// (001) BT” indicate the phase of the textured ceramics (T for tetragonal), the texturing direction [001], and the template used (BT).

Textured ceramics based on BT have emerged as highly promising lead-free piezoelectric materials, owing to their enhanced electromechanical properties achieved through controlled crystallographic orientation via TGG. Several templates, including BT [12,21,27,35,36,37], Ba_0.92_Ca_0.08_TiO_3_ (BCT) [38], SrTiO_3_ (ST) [39], BT + CaTiO_3_ (CT) [40], Ba_6_Ti1_7_O_40_ (B6T17) [41,42,43], and NN [44] templates, have been utilized in the texturing process. As illustrated in Figure 6a, (001)-oriented BT templates are frequently employed due to their strong compatibility with BT-based materials, with LF values ranging from 61% to 98% [12,21,27,35,36,37]. Furthermore, the use of ST and NN templates have demonstrated high degrees of orientation, with their LF values reaching 93% [39] and 95% [44], respectively. Similarly, BT + CT and BCT templates have been used, resulting in LF values of 83% [40] and 59% [38], respectively. B6T17 templates have been widely used in RTGG with reported LF values obtained between 50% and 80% [41,42,43]. These findings underscore the critical influence of the template selection in determining the crystallographic orientation degree (i.e., the LF) of BT-based textured ceramics, which is key to optimizing the material performance.

The piezoelectric performance of BT-based textured ceramics depends not only on the degree of the crystallographic orientation but also on the specific crystallographic direction and phase structure. Figure 6b shows the *d*_33_ values of various textured BT-based ceramics across different crystallographic phases, including the tetragonal (T), rhombohedral (R), and orthorhombic–tetragonal (O-T) phases. In particular, [001]-textured pure tetragonal BT using (001)-oriented BT templates exhibits *d*_33_ values ranging from 160 to 275 pC/N [21,27,35], while [110]-textured BT using (110)-oriented BT templates reach a *d*_33_ value of 788 pC/N [12], the highest reported for BT-based textured ceramics to date. This result highlights that the [110] texture direction is favorable for maximizing the piezoelectric performance. This is called an “engineering direction”, since it diverges from the spontaneous polarization direction (Ps ∥ [001]) of tetragonal BT and, hence, “engineers” an especially favorable domain configuration. It still relies on the same TGG method as that used for [001] texturing, just with (110)-oriented templates. In addition to textured pure BT ceramics, textured BCTZ-based systems demonstrate substantial potential, achieving *d*_33_ values from 462 to 755 pC/N when using (001)-oriented BT templates [14,36,37,45]. The highest *d*_33_ value of 755 pC/N was reported for O-phase-dominant BCTZ (Ps ∥ [011] and [101]) textured using (001)-oriented BT templates [14], where [001] texturing fosters a favorable domain configuration that enhances the extrinsic contributions alongside the intrinsic improvements from texturing. In summary, the optimal piezoelectric performance in BT-based textured ceramics is achieved by strategically aligning the crystallographic direction with the material’s intrinsic polarization pathways, leveraging both the phase structure and orientation to maximize the *d*_33_ values.

### 2.1. Template Chemistry Effect on Textured BaTiO_3_-Based Ceramics

#### 2.1.1. BT Templates for Texturing BT Ceramics

**Figure 7:**
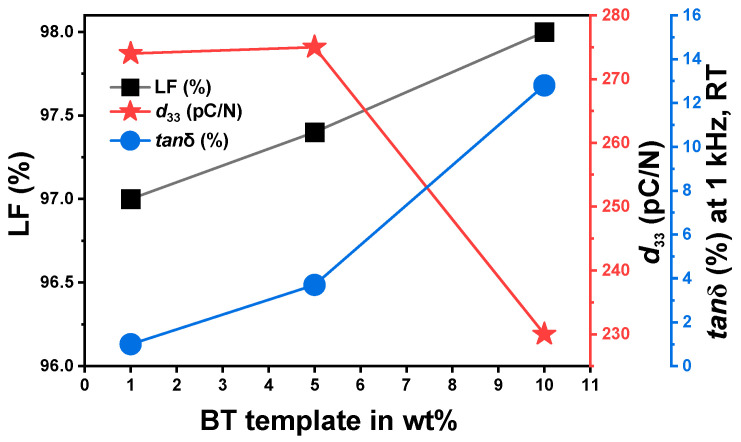
BT template amount versus Lotgering factor (LF), piezoelectric charge coefficient (*d*_33_), and dielectric loss (*tan*δ) in textured BT ceramics [21,27,35].

The use of BT templates in texturing BT-based ceramics offers benefits due to the identical structures and compositions between the template and the matrix. The chemical purity of BT templates is crucial to optimize for the right balance between the texture quality and resulting piezoelectric and dielectric properties [21,27,35]. Vriami et al. [21] reported that the residual Bi content in synthesized BT templates, obtained from a Bi_4_Ti_3_O_12_ precursor template, can range from 1.3% to 8% by weight. Furthermore, the risk of Bi diffusion into the matrix during sintering increases with higher template concentrations and can adversely affect the material properties. For instance, as illustrated in Figure 7, when the BT template amount increased from 1 wt% to 10 wt%, a modest rise in the LF was observed, but this was accompanied by a substantial rise in the dielectric loss (defined as the ratio of the imaginary to the real dielectric permittivity, also described as *tan*δ, where *δ* is the loss angle) from 1% to 13%, and a marked decline in the *d*_33_. These results suggest that while higher template concentrations enhance the texture, amounts above 5 wt% can introduce elevated dielectric losses and reduced piezoelectric performances, potentially linked to impurities from the template [21,27,35].

#### 2.1.2. BT Templates for Texturing BCT Ceramics

Recent advancements in the texturing of Ba_1−x_Ca_x_TiO_3_ (BCT) ceramics have demonstrated significant enhancements in their functional properties. Haugen et al. [46] reported a high degree of texture (99% LF) along the [001] orientation in BCT ceramics using 10 wt.% BT templates, yielding a *d*_33_ that was 207 pC/N higher than that of the nontextured counterpart, exhibiting a *d*_33_ of 155 pC/N. In contrast, Schultheiß et al. [40] used a combination of BT and CaTiO_3_ (CT) templates to texture BCT ceramics. While the CT templates raised the *T*_C_ from 92 °C to 117 °C by stabilizing the tetragonal phase, they also induced Ba/Ca concentration gradients near the grain boundaries due to lattice mismatches with the BCT matrix, which led to inhomogeneous regions and potential phase inconsistencies. The structural compatibility between the orthorhombic CT and tetragonal BCT likely introduced internal stresses, in contrast to the smoother grain growth observed with the BT template alone [40,46].

#### 2.1.3. BT Templates for Texturing BCTZ Ceramics

**Figure 8:**
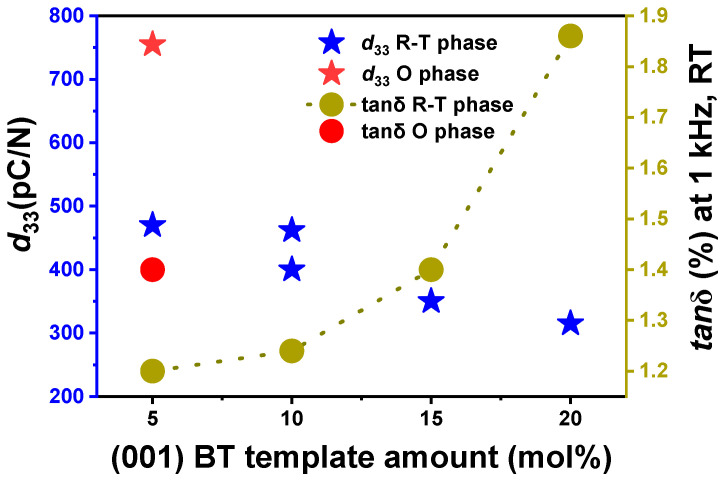
Piezoelectric coefficient (*d*_33_) and dielectric loss (*tan*δ) as a function of (001) BT template content (mol%) for both R-T and O phases in textured BCTZ ceramics, measured at room temperature [14,36,37].

(Ba,Ca)(Zr,Ti)O_3_ (BCTZ) ceramics are commonly textured using (001)-oriented BT platelet templates, with numerous studies highlighting the template amount as a decisive factor influencing not only the texture quality but also the resulting phase structure and electromechanical properties [14,36,37,45]. Figure 8 summarizes the impact of the BT template amount on textured BCTZ ceramics properties, highlighting that adding 5 mol% BT yields the highest electromechanical performance in both O-phase-dominant and R-T-phase textured BCTZ ceramics. For example, Ye et al. [36] investigated BT (T-phase) template amounts ranging from 5 to 20 mol% in R-T-phase BCTZ ceramics, identifying 5 mol% as optimal, achieving a moderate [001] orientation of LF = 82% and a *d*_33_ of 470 pC/N, along with pronounced tetragonality due to the T-phase BT and an increase in the *T*_C_ from 60 to 78 °C. In contrast, a higher amount than 5 mol% resulted in the templates overlapping and colliding during the alignment, reducing the texture quality and electromechanical properties. A related study by Bai et al. [37] used 10 mol% BT templates to texture R-T-phase BCTZ ceramics, resulting in a slightly lower LF (70%) and a *d*_33_ of 462 pC/N, with a decrease in the *T*_C_ from ~95 °C to 87 °C, ascribed to minor interdiffusion between the template and the matrix, likely associated with the higher template content than that used by Ye et al. In the most notable case, Liu et al. [14] reported [001]-textured BCZT with a dominantly O-phase ceramic using 5 mol% BT templates, achieving a high LF of 98.6% and a *d*_33_ of 755 pC/N. Here, the BT template helped optimize the electromechanical performance not only by promoting texturing but also by causing interfacial stress that refined the domain size, contributing to the enhanced piezoelectric properties. The observations summarized in Figure 8 illustrate that the 5 mol% BT template amount provides an optimal balance for texturing BCZT ceramics. A higher amount not only reduces the texture quality but also increases the dielectric loss (*tan*δ), pointing to a trade-off between the piezoelectric and dielectric properties. In summary, the BT template addition in BCTZ ceramics provides good texture quality, introduces internal stress, induces tetragonality, and excessive amounts may reduce the *T*_C_ and affect the electrical properties.

#### 2.1.4. ST Template in Texturing BZT and BST Ceramics

SrTiO_3_ (ST) templates have proven effective for obtaining well-textured structures with enhanced electromechanical properties, particularly in BT-based systems, such as Ba(Zr,Ti)O_3_ (BZT) and Ba(Sr,Ti)O_3_ (BST) ceramics [39,47]. Edward et al. [47] explored the use of (001)-oriented ST templates in BZT ceramics, adding 5 vol% ST to the BZT matrix, achieving an LF of 93% and a piezoelectric strain coefficient (*d*_33_*) of 975 pm/V under 5 kV/cm, which was approximately three times higher than that of randomly oriented BZT ceramics. However, the addition of ST templates reduced the dielectric permittivity, and the rhombohedral phase appeared to destabilize. This is likely due to local field concentrations around the ST inclusions, which acted as non-piezoelectric clamping points. These effects can constrain the domain wall motion, limiting the extrinsic contributions to the piezoelectric response. In a parallel study, Xue et at. [39] used 5 mol% ST templates for BST ceramics. As with BZT, the ST templates in the BST ceramics achieved a high degree of crystallographic orientation, with an LF of 93%, confirming a well-aligned [001] texture. The textured BST ceramics exhibited enhanced dielectric tunability (45.9%) compared to the randomly oriented ceramics (36.7%). However, the ST addition led to a slight decrease in the *T*_C_, which was attributed not to the texturing but to the compositional changes associated with the incorporation of Sr^2+^ ions from the ST templates into the Ba site of the BST lattice during sintering. In summary, applying ST templates ensured good textures in BZT and BST ceramics, achieving enhanced piezoelectric and dielectric tunability. However, challenges due to local compositional changes associated with the templates being chemically unstable need to be addressed.

#### 2.1.5. NN Template in Texturing BT Ceramics

Kamlo et al. [44] reported [111]-textured BT ceramics using NN templates, achieving an LF of 95%. Notably, the addition of 8 vol% NN templates transformed the conventional tetragonal phase into a cubic phase through the formation of a solid solution with the BT matrix. This phase change leads to paraelectric behavior. Although this structural transformation results in a lower relative permittivity than that of pure tetragonal BT, it also decreases the dielectric loss and improves the thermal stability.

#### 2.1.6. Summary of Template Chemistry Effects in BT-Based Ceramics

To briefly summarize the different template chemistries discussed in Section 2.1.1, Section 2.1.2, Section 2.1.3, Section 2.1.4 and Section 2.1.5, we can say that each template composition studied for textured BT contributes unique advantages and challenges. BT templates, widely used for BT, BCT, and BCTZ ceramics, offer excellent structural compatibility and high texture quality. However, excessive template amounts, above 5 mol%, can introduce impurities, reducing the piezoelectric performance and increasing the dielectric losses. Similarly, ST templates are effective at obtaining good texture quality and dielectric tunability in BZT and BST ceramics but may induce local compositional changes leading to the clamping effect that lowers the performance. Both BT and ST templates were used to obtain [001]-oriented ceramics; in contrast, NN templates were used to induce [111] texture.

### 2.2. Effect of Texture Direction in BaTiO_3_-Based Ceramics

Optimizing the texture direction in BT-based ceramics enables an enhanced piezoelectric performance through an engineered domain structure [12,46]. Randomly oriented BT ceramics have been shown to achieve high *d*_33_ values (e.g., 350 pC/N [48] and 460 pC/N [49]), attributed primarily to the refinement of domain structures through grain size reduction [12]. However, achieving fine grains in textured ceramics remains challenging due to the large template particles—generally between 5 and 15 µm—which cause large, final grains. One promising approach for refining domain structures, even with larger grain sizes, involves texture for domain engineering. Here, the grains are oriented away from the P_s_, in this case, the [110] and [111] directions [12]. Wada et al. [12] reported a *d*_33_ of 788 pC/N in [110]-oriented BT ceramics, the highest reported in the textured BT family. This large performance improvement is attributed to the engineered domain configuration with a high domain wall density and average domain size of only 800 nm, despite the grain size reaching 75 µm. The increased ferroelectric response in the [110]-textured BT is in line with predictions from theory. The directional dependence of the magnitude of the intrinsic piezoelectric response was outlined by Davies et al. [50]. They introduced the terms “extender” and” rotator” ferroelectrics for materials with maximum responses parallel to or rotated away from the polar axis, respectively. Room-temperature BT, which is in the tetragonal phase but close to the transition to the orthorhombic phase around 0 °C, falls into the rotator category, such that a non-[001] texture should be beneficial.

Haugen et al. [46] experimentally verified this directional dependence of the texture by investigating tetragonal Ba_0.92_Ca_0.08_TiO_3_ (BCT) ceramics using both (001) and (111) BT templates. The addition of Ca decreased the T-O-phase transition such that the BCT at room temperature was far from its orthorhombic state and became an extender ferroelectric. Their work demonstrated that [001]-textured BCT ceramics exhibit enhanced *d*_33_ values, reaching up to 207 pC/N, compared to 150 pC/N for untextured BCT. This performance was attributed to the alignment of the electric field with the polar axis, leveraging BCT’s extender ferroelectric nature. In contrast, [111]-textured BCT ceramics exhibited lower piezoelectric performances, with *d*_33_ values of 113 pC/N, due to the electric field being applied parallel to the non-polar axis. Haugen’s work also revealed that the [001] texture provided better temperature stability due to the alignment along the polar axis, where the piezoelectric response (*d*_33_) coefficient showed less sensitivity to temperature variations in the tetragonal phase. Future research will likely focus on texturing with templates that promote engineered domain configurations and grain miniaturization further to push the performance boundaries of BT-based textured ceramics.

## 3. Textured K_0.5_Na_0.5_NbO_3_-Based Ceramics

**Figure 9:**
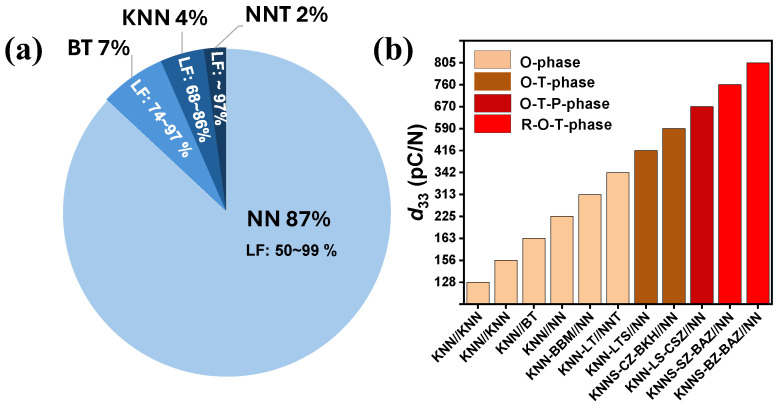
(**a**) Template distribution in textured KNN-based ceramics. The LF represents the Lotgering factor, the degree of texture in percent. (**b**) Piezoelectric charge coefficient (*d*_33_) values of various textured KNN-based ceramics across different crystallographic phases, including orthorhombic (O), orthorhombic–tetragonal (O-T), rhombohedral–orthorhombic–pseudocubic (O-T-P), and rhombohedral–orthorhombic–tetragonal (R-O-T) phases. All systems were textured along the [001] direction.

Various templates have been employed to optimize the texture in KNN-based ceramics, including NN [13,29,51,52,53,54,55,56,57,58,59,60,61,62,63,64,65,66,67,68,69,70,71,72,73,74,75,76,77,78,79,80,81,82,83,84,85,86,87,88,89], KNN [18,90], BT [28,53,91], and NaNb_0.8_Ta_0.2_O_3_ (NNT) [92]. As shown in Figure 9a, NN templates are by far the most used, with LF values ranging from 50% to 99%. In comparison, textured KNN-based ceramics using KNN, BT, and NNT templates achieved relatively lower LF values of 68–86%, 74–97%, and approximately 97%, respectively. Overall, the distribution of the templates and their respective LF values highlight the predominant use of NN, while KNN, BT, and NNT templates each present specific advantages that merit further exploration.

Figure 9b illustrates the reported *d*_33_ values of various textured KNN-based ceramics across different crystallographic phases, including the O, O-T, O-T-P, and R-O-T phases. The large number of phases studied comes from the tendency to engineer KNN-based systems (and other piezoelectric systems) with polymorphic or morphotropic phase boundaries, where the energy barrier for polarization reorientation is lower [4]. This explains why the largest d_33_ is found in multi-phase systems. All systems were textured along the [001] direction. Among the reported pure KNN ceramics, the templates textured with NN [29] in the O phase demonstrated a higher piezoelectric performance (225 pC/N) compared to those textured with KNN [18,90] and BT [28] (156 pC/N and 163 pC/N, respectively). Similarly, textured KNN-based ceramics using the NN template in the O-T phase exhibited *d*_33_ values up to 590 pC/N [54,89], those in the O-T-P phase exceeded 650 pC/N [57], and those using the NN template in the R-O-T phase achieved the highest reported *d*_33_ value of 805 pC/N [51,56]. This underscores the extensive investigation of the NN template across a wide range of KNN-based ceramics, with varying properties associated with the phase structure of the target material. Despite the antiferroelectric nature of NN, its use and effectiveness prove that it is a suitable choice for texturing KNN-based systems, showing excellent compatibility to achieve optimal properties.

**Figure 10:**
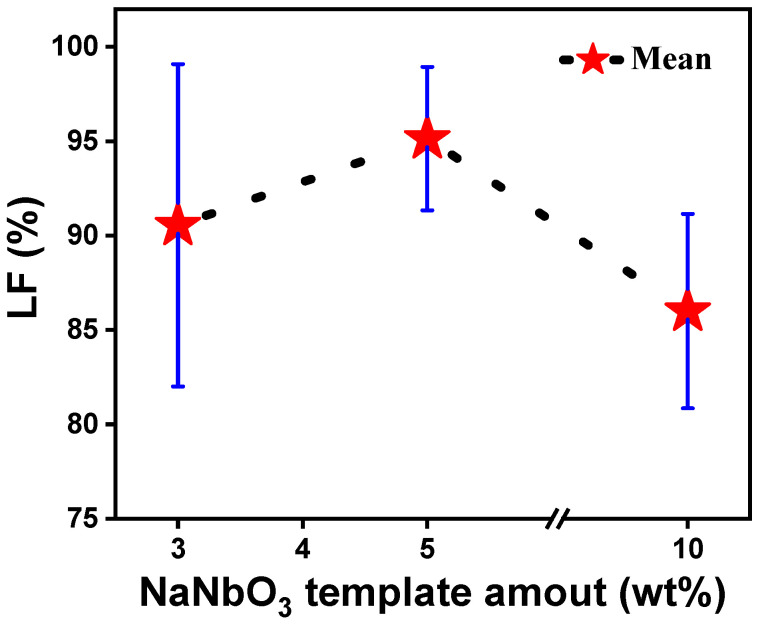
Lotgering factor (LF) as a function of NN template amount (wt%) in KNN-based ceramics. The values and error bars are based on data from various studies [13,29,51,52,53,54,55,56,57,58,59,60,61,62,63,64,65,66,67,68,69,70,71,72,73,74,75,76,77,78,79,80,81,82,83,84,85,86,87,88,89].

As NN is the most widely used template in KNN-based ceramics, we explored the optimal amount of NN for maximizing the LF. Figure 10 further illustrates a consistent trend across various studies: The 5 wt% NN templates yielded the highest LFs, consistently achieving LF values exceeding 90%, compared to the larger disparity observed when using 3 wt% and 10 wt%. This suggests that 5 wt% NN effectively balances the texture quality with the maintenance of reproducibility, making it the recommended amount for optimal texturing in KNN-based ceramics. The reduction in the LF with higher template contents can be related to template–template overlap, which impedes alignment, as well as local compositional inhomogeneities that reduce the templated grain growth.

### 3.1. Template Chemistry Effect on Textured KNN-Based Ceramics

#### 3.1.1. NN Templates for Texturing KNN-Based Ceramics

NN templates are pivotal in texturing KNN-based ceramics, largely due to their structural and compositional compatibility with the KNN matrix. These templates are widely used during the RTGG process, where K^+^ ions diffuse into the NN templates, while Na^+^ ions migrate out into the matrix during the heat treatment process, facilitating the formation of stoichiometric KNN [82,85,87]. The completion of this reaction is essential, as it directly impacts the final composition and properties of the ceramics. Several studies have utilized energy dispersive spectroscopy (EDS) to investigate the completion of the reaction between the template and the matrix based on the presence or absence of a compositional gradient in the textured grain [82,85,87]. However, the resolution limitations of EDS mean that this conclusion is not definitive [92]. To address these uncertainties, Tutuncu et al. conducted in situ X-ray diffraction (XRD), which provided more detailed insights. Their findings revealed that NN templates undergo a chemical transformation into the KNN matrix, followed by grain growth through homoepitaxial templating, indicating a near-complete transformation of the NN templates [93]. Additionally, other reports have indicated that NN templates react and dissolve into the matrix during sintering, forming rectangular holes in the areas where NN templates existed, as confirmed by the microstructure [51,63,94]. In conclusion, NN templates play a crucial role in the texturing of KNN-based ceramics during RTGG, with in situ XRD confirming their near-complete chemical transformation into the matrix. However, the formation of voids in the microstructure, where NN templates once existed, raises concerns about its potential impact on the electromechanical properties of the textured ceramics. Future research on removing these voids through advanced densification techniques or optimizing the sintering process could help to fully realize the performance of KNN-based textured ceramics.

#### 3.1.2. KNN Templates for Texturing KNN-Based Ceramics

Unlike NN templates, which can lead to void formation and residual defects in the microstructure after reacting with the KNN matrix, KNN templates offer a more reliable solution due to their chemical and structural compatibility with the matrix. This compatibility enables homoepitaxial grain growth without requiring a chemical reaction between the template and the matrix, depending on the matrix composition. As a result, the risk of incomplete reactions and microstructural defects commonly associated with NN templates is minimized. Haugen et al. [18] demonstrated the successful use of [001] needle-like KNN templates to texture pure KNN ceramics, achieving an LF of 86% and a *d*_33_ of 125 pC/N. The strong alignment of the grains contributed to an enhanced non-180° domain reorientation, improving the electromechanical properties of the material. In a related study, Liu et al. [90] introduced strontium (Sr) into the KNN template synthesis through a heterogeneous microcrystalline transformation process, also used for texturing pure KNN ceramics, achieving an LF of 68%. Adding Sr helped stabilize the tungsten bronze precursor (K_2_Nb_8_O_21_), from which the needle-like KNN templates were synthesized. Sr also enhanced the densification by promoting the formation of low-energy grain boundaries. However, compared to the texture achieved using NN templates, where a *d*_33_ of 225 pC/N and an LF of 96.6% were reported [29], the results obtained with KNN templates are lower. This difference in performance could be attributed to the comparatively lower LF values of 86% and 68% in the studies using KNN templates. While NN templates have been extensively studied, the use of KNN templates remains less explored. The further investigation and optimization of texturing KNN ceramics using KNN templates would be a promising area for future research.

#### 3.1.3. BT Templates for Texturing KNN-Based Ceramics

The use of BT templates to texture KNN-based ceramics is less common, possibly due to the compositional and structural differences between the BT template and the KNN matrix. A key challenge in such hetero-templated grain growth is maintaining the template stability during sintering to avoid altering the final composition of the textured ceramics [28,53]. Lv et al. [28] demonstrated that employing (001)-oriented BT templates for texturing in KNN ceramics achieved significant texturing levels but resulted in a notable drop in the *T*_C_ from 420 °C to 320 °C, possibly due to the formation of a solid solution between the BT templates and the KNN matrix. Park et al. [91] introduced (001)-oriented heterostructured BT (h-BT) templates to mitigate these issues, where KNN nanoparticles were nucleated onto BT templates. This pre-synthesized template helped to reduce the interfacial strain and improve the grain growth, resulting in a better texture quality compared to that of bare BT templates. The phase transition temperatures (*T*_o-t_ at 151 °C, *T*_C_ at 390 °C) in h-BT-KNN textured ceramics were nearly identical to those in nontextured ceramics, indicating that the h-BT templates minimized the compositional changes and lattice strain, preserving the phase stability.

#### 3.1.4. Summary of Template Chemistry Effects on Textured KNN

NN templates are by far the most studied and provide a high degree of texture and a good piezoelectric response. Their main challenge is the formation of voids in the matrix structure as the template diffuses into the material during TGG. BT templates are less suitable, due to the larger compositional difference. While the availability of KNN templates is limited, good results have been obtained with KNN needle-shaped templates, where both compositional differences and voids after dissolution can be avoided.

### 3.2. Effect of Texture Direction in KNN-Based Ceramics

KNN ceramics of various phases, such as O-phase [18,28,29,90,92], O-T-phase [54,89], O-T-P-phase [57], and R-O-T-phase [51,56] ceramics, have been successfully textured. All studies used [001] as the direction of texture, which is a non-polar axis for all phases except the T phase. This specific alignment allows for domain engineering to strategically optimize the domain contributions, enhancing the piezoelectric responses by capitalizing on the polarization directions characteristic of each phase. While detailed data are unavailable for KNN- or KNN-based compositions, pure KNbO_3_ is known as a rotator ferroelectric [50] such that the same can be expected for KNN-based compositions isostructural to KNbO_3_.

In the O phase, the P_s_ vector is along the [101] direction. Texture aligned along the non-polar [001] direction is therefore beneficial for the assumed rotator ferroelectricity and enables non-180° domain switching [18]. However, the *d*_33_ values of O-phase KNN are generally low, ranging from 128 pC/N to 342 pC/N [62].

In the O-T phase, [001] textured ceramics show *d*_33_ values ranging from 416 to 590 pC/N, which are higher than those in textured O-phase KNN ceramics. O-T-phase KNN-Ta-based ceramics with the [001] texture promote the formation of diverse large-scale domain morphologies, such as 180° stripe-shaped, 45° fish-bone, and extensive 90° labyrinth domains [62], distinct from the smaller nano-microdomains in random ceramics. In addition, the long-range ordered domains and poling pattern stability at elevated temperatures confer enhanced thermal stability on the material. This orientation effect aligns with Saito et al. [89]’s early findings on [001] texturing in the O-T phase of KNN-based ceramics, where the temperature-independent strain was attributed to changes in the amplitude and balance between two strain components: one from lattice motion and the other from domain wall motion.

In O-T-P-phase KNN ceramics, the [001] texture has a pronounced effect on the domain structure and piezoelectric properties. Go et al. [57] observed that the [001] orientation in O-T-P-phase KNN-based compositions promotes the formation of a complex, multi-scale domain structure that includes stable, large-scale domains interspersed with smaller nanodomains. These nanoscale domains act as transitional zones, reducing the boundary energy and enabling smoother domain rotation and switching under an electric field. This hierarchical arrangement, characterized by both large, long-range domains and interstitial nanodomains facilitates polarization rotation, which helps to maintain high *d*_33_ values (up to 670 pC/N) and electrical stability over a wide temperature range. This structured domain hierarchy mirrors the stabilization effects noted in Lin et al.’s O-T-phase textured KNN-Ta ceramics. The inclusion of the pseudocubic phase in O-T-P-phase compositions acts as a buffer that further stabilizes these domain arrangements.

In R-O-T-phase KNN-based textured ceramics, optimizing the ratio of the R, O, and T structures along the [001] crystallographic direction enables a superior piezoelectric performance due to enhanced domain contributions. Go et al. [51] report a record-high *d*_33_ (805 pC/N) in NKNS-0.02BZ-0.02BAZ textured ceramics with an R-O-T structure containing an approximately 80% R-O phase. This high R-O fraction ensures a majority of domains with the [110] and [111] polarization directions, thereby facilitating effective polarization rotation and domain reorientation under external electric fields. Thus, Go et al. conclude that domain engineering alone does not entirely explain the increase in the *d*_33_ values. Instead, the synergy of domain and composition engineering in achieving a balanced R-O-T structure with a high R-O-phase fraction is essential for superior piezoelectricity in [001]-textured R-O-T-phase KNN-based ceramics.

## 4. Textured Na_0.5_Bi_0.5_TiO_3_-Based Ceramics

**Figure 11:**
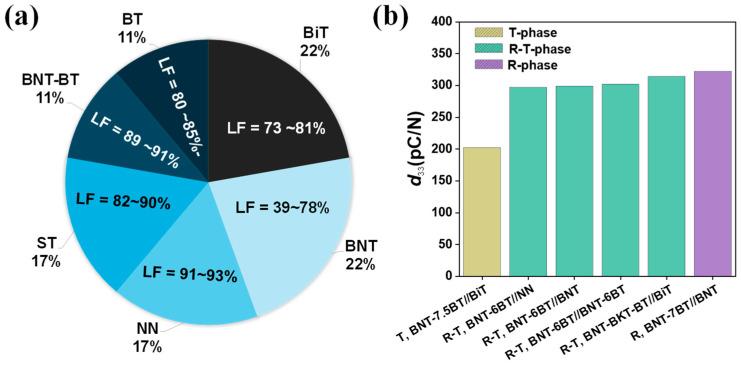
(**a**) Template distribution in textured NBT-based ceramics, showing the Lotgering factor (LF) values for various templates. (**b**) Piezoelectric charge coefficient (*d*_33_) values of various textured BNT-based ceramics across different crystallographic phases, including tetragonal (T), rhombohedral–tetragonal (R-T), and rhombohedral (R) phases. All textured along the [001] direction.

BNT-based ceramics are considered as promising lead-free piezoelectrics due to their good electromechanical properties, and texturing is becoming an increasingly popular direction to further improve their performance. Several templates, including Bi_4_Ti_3_O_12_ (BiT) [95,96,97,98], NN [95,96,97], BT [98,99], ST [100,101,102], BNT [30,103,104,105], and binary combinations such as BNT-BT [32,106], have been utilized to texture BNT systems. As shown in Figure 11a, the BNT template is the most extensively used for texturing BNT systems, but with a broad LF range from 39% to 78%, highlighting the variability in texturing outcomes. In contrast, BiT, NN, BT, ST, and BNT-BT templates exhibit higher LF values of 73% to 80%, 91% to 93%, 80% to 85%, 82% to 90%, and 89% to 91%, respectively. These LF variations across template types emphasize the critical role of the template selection in optimizing the crystallographic orientation in NBT-based ceramics.

In the (1−x)Bi_0.5_Na_0.5_TiO_3−x_BT system, an MPB (morphotropic phase boundary) lies within 0.06 ≤ x ≤ 0.07, where both the rhombohedral (R) and tetragonal (T) phases coexist [107,108,109,110]. Among textured BNT-based systems, BNT-6BT ceramics, located at the MPB (R-T phase), have been textured with various templates, including NN, BNT, and BNT-6BT, exhibiting nearly identical *d*_33_ values of 297, 299, and 302 pC/N, respectively, as shown in Figure 11b [30,31,32]. BNT templates, as one of the most widely used options, have also been employed to texture various compositions: BNT-5BT, BNT-6BT, and BNT-7BT ceramics, which exhibit *d*_33_ values of 245, 297, and 322 pC/N, respectively [30,105,111], which indicates that BNT templates are effective at improving and adjusting the piezoelectric properties of BNT-based ceramics, with the *d*_33_ values increasing with the BT content near the MPB.

### 4.1. Template Chemistry Effect on Textured BNT-Based Ceramics

#### 4.1.1. BiT Templates for Texturing BNT-Based Ceramics

Bi_4_Ti_3_O_12_ (BiT) templates are frequently utilized for RTGG, where the template reacts with the BNT complementary matrix during calcination. Kimura et al. [112] studied textured BNT-6BT ceramics with varying BiT template amounts and observed that a 20% BiT content, providing 20% of Ti^4+^ and 53.3% of Bi^3+^ in the matrix, resulted in a relatively low LF of 36%. By increasing the template content to 37.5%, supplying 37.5% Ti^4+^ and 100% Bi^3+^, the LF improved to 70% at the same sintering temperature. Later, Chen et al. [113] achieved a higher LF of 73.5% with a lower BiT template amount of 7.7 mol% in textured BNT-7.5BT ceramics. Similarly, Zhao et al. [114] reported LF values of 95% for BNKT ceramics textured with 12.5 mol% BiT, highlighting that Na^+^ and K^+^ diffuse into BiT, while Bi^3+^ diffuses out. Additionally, BiT templates have been used to texture BNT-BKT-BT ceramics, achieving an LF of 81% with 12 mol% templates [115]. These findings underscore the critical role of the amount of BiT templates in determining the degree of texture, with the LF values varying from 36% to 95%.

#### 4.1.2. BT Templates for Texturing BNT-Based Ceramics

The study by Bai et al. [99] explores the effects of different templates, including BT, ST, and NN, on [00l]-textured 83BNT-17BKT ceramics. Unlike ST and NN, the introduction of BT templates significantly lowered the *T*_F-R_ (ferroelectric–relaxor-phase transformation temperature) below room temperature. This suppression of the long-range ferroelectric order enhanced the electric-field-induced response, thereby contributing to improved temperature stability. Similarly, Zhang et al. [98] investigated [001]-textured 76BNT-24ST ceramics using 5 wt% BT templates, achieving an LF of 86%. However, the BT templates were found to partially integrate into the ceramic matrix during sintering. This integration likely changed the matrix composition, leading to shifts in the *T*_F-R_ to lower temperatures and the *T*_m_ (temperature corresponding to the maximum dielectric constant) to higher temperatures. Besides facilitating the formation of textured structures, the use of BT templates has been shown to shift the *T*_F-R_ in both BNT-BKT and BNT-ST systems, influencing the phase transition behavior and thermal stability.

#### 4.1.3. ST Template in Texturing BNT-Based Ceramics

The use of ST templates in BNT-based ceramics has shown promise. Bai et al. [100] investigated the effect of the ST template on textured BNT-BKT ceramics, achieving an LF of 90%. However, much like BT templates, the introduction of ST templates lowered the *T*_F-R_, favoring a relaxor-like state at lower temperatures. This shift in the phase behavior resulted in improved strain responses, with a large strain of 0.38% observed at 15 mol% ST, but at the cost of reduced thermal stability. Bai et al. [101] also investigated the effect of ST templates on textured BNT-ST ceramics by varying the template content from 0, 5, and 10 to 20 wt%, achieving an LF of 82% at 10 wt% ST. However, when the template content increased above 10 wt%, the LF declined. This decrease was attributed to increased template crowding, where overlap and collision among ST template particles disrupted the alignment, thereby reducing the texturing efficiency.

Similar to previous observations with BT templates, introducing ST templates into both BNT-BKT and BNT-ST lowered the depolarization temperature (*T*_d_), shifting the material behavior toward a relaxor state. This shift is attributed to interdiffusion between the ST templates and the BNT matrix, which introduces internal stresses. These effects disrupt the long-range ferroelectric ordering and encourage a short-range relaxor state, enhancing the strain responses but sacrificing the thermal stability. Bai et al. noted a strain response improvement in both material systems, demonstrating the potential of ST templates to enhance the electromechanical performance in BNT-based ceramics. However, the trade-off in the thermal stability underscores the need for the careful selection and optimization of the template content when targeting specific applications in textured BNT-based ceramics.

#### 4.1.4. BNT Template in Texturing BNT-Based Ceramics

Compared to BT templates, BNT templates are favorable for texturing BNT-based systems due to their closer compositional match with the matrix. Hussain et al. [103] reported that textured 0.94BNT-0.06BZ ceramics exhibited an LF of 80% with a 15 wt% BNT template addition. Notably, the aligned grain structure induced by BNT templates stabilizes the ferroelectric phase, reducing the relaxor behavior and encouraging a stable response under electric fields. Similarly, Zhang et al. [104] reported similar findings in BNT–BT–3AN ceramics, observing an LF of 89%. Textured samples provided a high unipolar strain of 0.38% at 5 kV/mm, which is 78% higher than the values of the randomly oriented ones. The enhanced electric-field-induced strain at the relatively lower field was attributed primarily to the facilitated phase transition to a long-range ordered ferroelectric phase along the [001] direction. In contrast to the use of other templates like BT and ST, textured BNT-based ceramics using BNT templates promote the long-range ferroelectric order stabilizing effect on the ferroelectric phase.

#### 4.1.5. BNT-BT Template in Texturing BNT-Based Ceramics

BNT-BT templates compositionally identical to the BNT-BT matrix have been reported to achieve high-quality texturing with better thermal stability [32]. This close chemical match enables a homoepitaxial growth process during TGG, allowing the template to integrate seamlessly without introducing misfit strain or internal stresses at the crystallization interface. Ma et al. [32] observed that in [001]-textured 0.94BNT-0.06BT ceramics, using 0.94BNT-0.06BT templates achieved a high LF of 91%. This stress-free growth is evidenced by the absence of any observable shifts or changes in the XRD peak position, underscoring the structural coherence between the template and matrix and enabling enhanced crystallographic texture. The introduction of [001] texture through homoepitaxial TGG also slightly increases the *T*_F-R_, rising from 98 °C in nontextured samples to 106 °C in textured ceramics.

#### 4.1.6. Summary of Template Chemistry Effects in BNT-BT

**Figure 12:**
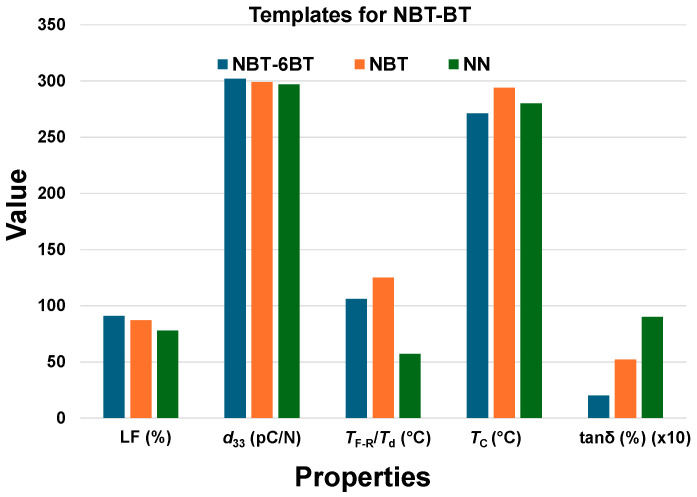
Comparative analysis of Lotgering factor (LF), piezoelectric charge coefficient (*d*_33_), depolarization temperature (*T*_d_), and dielectric loss (*tan*δ) in BNT-6BT ceramics textured with different templates (NBT-6BT, NBT, and NN). To improve visibility, *tan*δ (%) values were multiplied by 10.

The use of varying templates for BNT-6BT ceramics illustrates that the template–matrix chemical compatibility is crucial for optimizing the thermal and dielectric properties. As shown in Figure 12, the LF slightly decreased from 91%, 87%, and 78% as the compositional similarity widened from the BNT-6BT, NBT and NN templates, respectively. However, the obtained *d*_33_ values are nearly the same regardless of the templates used. Nevertheless, significant distinctions are observed in the *T*_d_ and *tan*δ, where the template compatibility influences the thermal stability and energy efficiency. Notably, when NN [30] templates are used, a low *T*_d_ of 57 °C and a high *tan*δ of 9% were reported, suggesting that limited chemical compatibility may lead to increased dielectric losses and reduced thermal stability due to internal stresses at the template–matrix interface. In contrast, NBT [31] and NBT-6BT [32] templates, compositionally similar to BNT-6BT, enable seamless homoepitaxial grain growth, thereby reducing the interfacial stress and enhancing the thermal stability. A higher *T*_d_ of 140 °C with a lower *tan*δ of 5.2% were reported with NBT templates, while NBT-6BT templates achieved an optimal *T*_d_ of 106 °C, with a minimal *tan*δ of 2% reported. These findings underscore the importance of compositional alignment in the template choice for BNT-based textured ceramics, with homoepitaxial growth providing a pathway to enhanced phase stability, improved thermal performance, and reduced dielectric losses.

### 4.2. Effect of Texture Direction in BNT-Based Ceramics

In [001]-textured BNT-BT-based ceramics, R-phase, T-phase, and near-to-MPB compositions have been textured. Fancher et al. [105] investigated textured R-phase BNT-5BT ceramics, reporting an enhanced *d*_33_ of 245 pC/N related to polarization rotation facilitated by a rhombohedral-to-monoclinic domain shift. In the T phase, textured BNT-7.5BT ceramics were found by Chen et al. [113] to have a *d*_33_ of 202 pC/N, attributed to the electric-field-induced lattice distortion along the [001] direction. In the case of textured BNT-6BT-based ceramics, where this composition lies near to the MPB, Bai et al. [30] achieved a high *d*_33_ of 297 pC/N, nearly double the 151 pC/N of the randomly oriented counterparts, primarily attributed to the non-180° domain switching. These findings demonstrate that [001] texturing enhances the *d*_33_ in BNT-BT ceramics, with phase-specific mechanisms: polarization rotation in the R phase, lattice distortion in the T phase, and non-180° domain switching near the MPB.

The [001]-texturing direction not only enhances the piezoelectric properties but also influences the thermal stability. In Maurya et al. [111]’s study, although the highest piezoelectric response (*d*_33_) value of 322 pC/N was achieved in the [001]-oriented NBT-7BT (the MPB phase, close to the T phase) ceramics, the *T*_d_ dropped significantly from 130 °C to 90 °C post-texturing, attributed to the relaxor nature, which led to earlier depolarization. In contrast, the randomly oriented ceramics exhibited a higher *T*_d_ due to the stronger long-range ferroelectric order, suggesting that while texturing improves the piezoelectric response, it introduces a trade-off in the thermal stability. Contrary to Maurya et al.’s work, Chen et al. [113] and Bai et al. [30] observed that texturing could enhance the thermal stability. Chen et al. reported a *T*_d_ increase from 90 °C to 140 °C in [001]-textured BNT-7.5BT (T-phase) ceramics, attributing this to the reinforced long-range ferroelectric order due to a high (h00) ordered degree. Similarly, Bai et al. demonstrated a *T*_d_ rise from 32 °C to 57 °C in [001]-textured BNT-6BT (MPB-phase) ceramics, linking this improvement to the stabilization of the ferroelectric domains, achieving both high piezoelectricity and enhanced thermal stability.

BNT-based ceramics have also been textured to optimize their energy storage performances along both the [001] and [111] directions. Ji et al. [116] developed [001]-textured 0.7(0.99NBT-0.01BY)-0.3ST ceramics using (001)-oriented BNT templates, achieving an LF of 82%. These ceramics exhibited an energy storage density of 5.34 J/cm^3^ with an efficiency of 83.91%. In contrast, Chen et al. [117] fabricated [111]-textured 0.8(0.99NBT-0.01BY)-0.2ST ceramics using (111)-oriented BT templates, achieving an LF of 74%, an energy storage density of 3.26 J/cm^3^, and an efficiency of 76.3%. The performance differences might have originated from the slight compositional differences, template types, orientation directions, and degrees of texture. Notably, [111]-oriented ceramics exhibited reduced electric-field-induced strain due to the minimal electrostrictive effect in the [111] direction, resulting in an enhanced breakdown strength. Li et al. [118] extended the investigation to [111]-textured NBT-SBT multilayers, achieving an LF of 91% using (111)-oriented ST templates. These multilayers demonstrated a breakdown strength of 103 MV/m (a 65% increase compared to nontextured ceramics) and achieved an energy storage density of 21.5 J/cm^3^ with 80% efficiency. The substantially lowered strain improved the breakdown strength and reduced the failure probability, enhancing the structural reliability in the multilayer configuration. This underscores the potential of [111] orientation for applications requiring superior breakdown strength and better performance stability.

## 5. Effect of Template Morphology (Size and Shape) on Textured Ceramics

Independent of the material composition, the morphology of the templates plays a pivotal role in the TGG process, influencing the degree of the grain orientation, the driving force of the grain growth, and the final textured grain size [33,34,119,120,121,122]. The alignment of anisotropic template particles within a fine-grained matrix depends on the aspect ratio, size disparity between the templates and matrix particles, and template size distribution, each of which plays a critical role in achieving a high degree of texturing [121,122].

A higher aspect ratio of the template generally promotes better alignment within the matrix during the tape-casting process, as elongated templates tend to orient themselves effectively under the influence of shear forces [120,122]. Furthermore, a uniform size distribution of the templates enhances this alignment by minimizing irregularities during tape casting, especially since the process is conducted with a constant blade gap, which imposes a uniform thickness constraint on the slurry [6,34,120,121,122]. Beyond alignment, the growth of the template by consuming matrix components during sintering plays a critical role in achieving a high degree of texture. This TGG mechanism is strongly influenced by the size difference between the template and the matrix grains. A significant size disparity provides a larger driving force for TGG, facilitating preferential growth in the desired crystallographic direction. Additionally, the volume fraction of the template is a pivotal factor, as it determines the extent of the matrix consumed during TGG, ultimately influencing the size of the resulting textured grains. Uniform-sized templates, when well dispersed within the matrix, result in more uniform TGG grains, minimizing defects and promoting consistency in the grain alignment [15,120,121]. While large templates are beneficial for alignment, a consequence is large grains in the textured ceramic, which is well known to reduce the mechanical strength and can increase the domain size. Still, the texture has the possibility of providing small domains through domain engineering, as discussed in Section 2.2.

**Figure 13:**
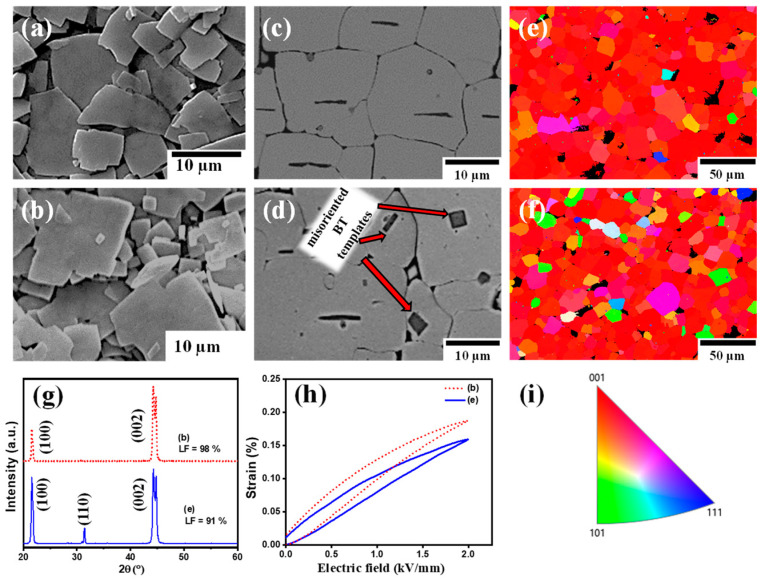
(**a**) BT templates with a narrow size distribution; (**b**) BT templates with a wide size distribution; (**c**) textured structure using (**a**); (**d**) textured structure using (**b**); (**e**) EBSD map of textured structure using (**a**); (**f**) EBSD map of textured structure using (**b**); (**g**) XRD patterns of textured ceramics and the degree of texture indicated by the Lotgering factor (LF); (**h**) strain versus electric-field curves; and (**i**) pole figure indicating grain orientation distribution. The [001] texture direction is vertical in Figure (**c**,**d**) and out of the plane in Figure (**e**,**f**). Figures are reprinted with permission from [34,123].

Figure 13 further illustrates these effects in textured PMN-PIN-PT ceramics prepared with a 3 vol% of BT templates [123]. In Figure 13a, the BT templates exhibit a narrow size distribution, leading to a well-aligned, textured structure with a uniform grain size, as evidenced in Figure 13c. In contrast, Figure 13b shows a broader size distribution, resulting in significant misalignments and a non-uniform grain structure, as highlighted in Figure 13d. Here, smaller templates, particularly those under 5 µm in size, appear misaligned, likely due to their inability to orient correctly during tape casting. The EBSD images in Figure 13e,f vividly depict these differences. In Figure 13e, the predominance of red pigmentation indicates a strong [001] grain orientation, reflecting a high degree of texture achieved with the narrow size distribution templates. Conversely, Figure 13f shows a wide array of colors, representing grains deviating from the preferred orientation due to the misalignment of smaller templates and a less uniform size distribution. This high degree of texture (an LF of 98%), achieved in the sample from Figure 13c, is observed in the XRD pattern in Figure 13g. Only the (100) and (200) peaks are visible in the sample in Figure 13c, compared to the additional (110) peak observed in the sample from Figure 13d, signifying improved texturing. This effective alignment translates to enhanced electromechanical properties, as demonstrated in Figure 13h, where the textured ceramic with well-aligned templates outperforms the less-textured counterpart. Therefore, the size distribution of the template needs to be carefully controlled to ensure optimal alignment and a high degree of texture. Narrow size distributions promote uniform grain growth and effective alignment during tape casting, as reflected in the superior texturing (an LF of 98%) and enhanced electromechanical properties observed in the sample with well-oriented templates. Conversely, broader size distributions lead to misaligned templates and deviations from the desired [001] orientation, resulting in a reduced texturing quality and compromised functional performance. To further enhance the density of sintered textured ceramics, optimizing the template alignment at a reduced template content can be effective. A lower template content minimizes the porosity while maintaining sufficient alignment to achieve a high degree of texturing. Additionally, the incorporation of sintering aids during the TGG process can promote densification by enhancing the mass transport and reducing grain boundary resistance, resulting in a denser final microstructure. These strategies, in combination with the careful control of the template morphology and distribution, offer a pathway to improve the density and performance of textured ceramics.

**Figure 14:**
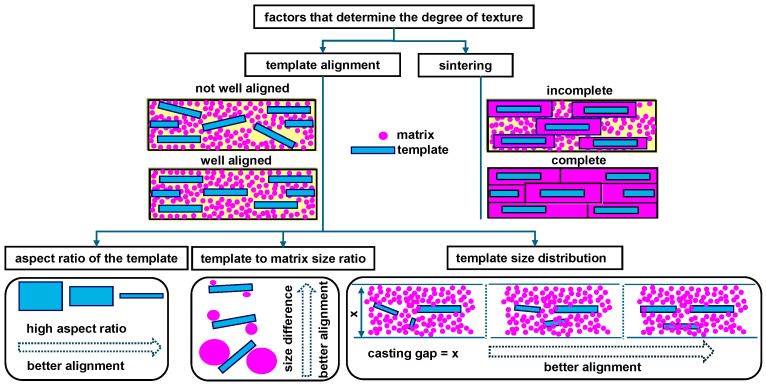
Influence of template morphology on the degree of texture in piezoelectric ceramics during the templated grain growth process when using plate-like templates aligned using the tape-casting process.

The accompanying Figure 14 elucidates these relationships, illustrating how the interplay of the aspect ratio, size disparity, and distribution in the template morphology orchestrates optimal alignment and texturing. Collectively, these insights underscore the importance of the template morphology as a fundamental design parameter, facilitating advances in textured ceramics with single-crystal-like properties, which are crucial for next-generation piezoelectric applications.

## 6. Conclusions

This review highlights the critical role of templates in the templated grain growth (TGG) of textured lead-free piezoelectric ceramics, specifically examining systems such as BaTiO_3_ (BT), (K_0.5_Na_0.5_)NbO_3_ (KNN), and Bi_0.5_Na_0.5_TiO_3_ (BNT). We emphasize how the template chemical composition, orientation, and morphology influence the crystallographic orientation, intrinsic properties, domain configurations, and phase structure of textured ceramics, all of which directly impact their piezoelectric and dielectric properties.

The templates’ chemical composition plays a crucial role. For all systems, a homo-templated grain growth system would be beneficial for obtaining chemically pure textured ceramics, without voids being formed by the templates dissolving. For NBT-BT, the use of NBT-BT templates is especially beneficial when it comes to retaining a high *T*_D_ and a low *tan*δ. Also, for BT-based ceramics, the highest degree of texture without clamping from residual templates or the formation of paraelectric phases is obtained with BT templates. For BT and BNT-BT, the homo-templated approach is common since both BT and BNT-BT platelet templates are readily available, while KNN templates are less available.

We also underscore the importance of selecting the optimal texturing directions, with the [001], [110], and [111] orientations explored across different systems. Among these, [001] texturing via tape casting is the most widely reported. However, depending on the target material’s phase structure and composition, it can be more beneficial with texture in a non-[001] direction. The texture away from the spontaneous polarization vector shows a significantly enhanced performance by enabling domain engineering and ferroelectric rotator behavior. For example, in tetragonal-phase BT, [110] texturing nearly triples the piezoelectric charge coefficient compared to [001] texturing. This effect has similarly been observed in KNN- and BNT-based systems, reinforcing the significance of domain engineering for maximizing the piezoelectric properties through both intrinsic and extrinsic mechanisms.

Finally, we highlight the impact of the template morphology, where a large template size with a narrow size distribution and high aspect ratio is important for achieving the optimal alignment and crystallographic texture during TGG.

## Data Availability

No new data were created or analyzed in this study. Data sharing is not applicable to this article.
